# Echocardiographic assessment and percutaneous closure of multiple atrial septal defects

**DOI:** 10.1186/1476-7120-2-9

**Published:** 2004-07-21

**Authors:** Andrew RJ Mitchell, Philip Roberts, Jonas Eichhöfer, Jonathan Timperley, Oliver JM Ormerod

**Affiliations:** 1 The Department of Cardiology, John Radcliffe Hospital, Oxford, OX3 9DU, United Kingdom; 2 The Department of Paediatric Cardiology, John Radcliffe Hospital, Oxford, OX3 9DU, United Kingdom

**Keywords:** Atrial septal defects, Amplatzer septal occluder, echocardiography

## Abstract

Atrial septal defect closure is now routinely performed using a percutaneous approach under echocardiographic guidance. Centrally located, secundum defects are ideal for device closure but there is considerable morphological variation in size and location of the defects. A small proportion of atrial septal defects may have multiple fenestrations and these are often considered unsuitable for device closure. We report three cases of multiple atrial septal defects successfully closed with two Amplatzer septal occluders.

## Introduction

Atrial septal defect (ASD) closure is now commonly performed using a transcatheter, percutaneous approach and with the Amplatzer septal occluder, large defects can be safely closed [1,2]. Device deployment requires a rim of atrial septal tissue surrounding the defect to allow effective capture of the septum by the occluder. The rim of tissue is also important to separate the septal occluder from important structures including the inferior vena cava, coronary sinus and the atrioventricular valves.

The majority of patients require a single device for closure of the ASD but a small proportion of patients may have more than one defect in the atrial septum. This can be difficult to diagnose using transthoracic echocardiography (TTE) as abnormal colour flow obscures the origins of the shunt, particularly if the second defect is situated inferiorly. We report three cases of patients referred for ASD closures that were found to have multiple ASDs and the techniques used to close these defects.

### Case 1

A 34-year old woman was referred for consideration of percutaneous ASD closure. The ASD had been diagnosed when the patient was 12 years old and TTE had suggested that the right ventricle was dilating. At cardiac catheterisation there were mildly elevated right ventricular systolic pressures and a pulmonary to systemic flow ratio of over two. The secundum ASD was estimated to be 15 mm wide using TTE with aneurysmal formation of the interatrial septum. The patient was admitted for percutaneous ASD closure and underwent uncomplicated placement of a 17 mm Amplatzer septal occluder. Transesophageal echocardiography (TEE) during the procedure revealed the presence of a second ASD near the inferior vena cava and a small post-procedure shunt. The septal occluder did not completely cover both defects. Equivalent chest x-ray radiation dose (assuming a single posteroanterior projection chest x-ray is eight centi-Gray/cm^2^) was 400. Repeat TTE continued to demonstrate left to right shunting and the patient was readmitted for a further device closure six months later. At cardiac catheterisation, the second defect was identified low in the secundum septum between the fossa ovalis and the mouth of the coronary sinus. The defect was successfully closed with a 9 mm Amplatzer septal occluder with no evidence of obstructed flow in either the coronary sinus or the inferior vena cava. Equivalent chest x-ray radiation dose for the second procedure was 187. The patient remained well with no evidence of residual shunt six months following the procedure.

### Case 2

**Figure 1:**
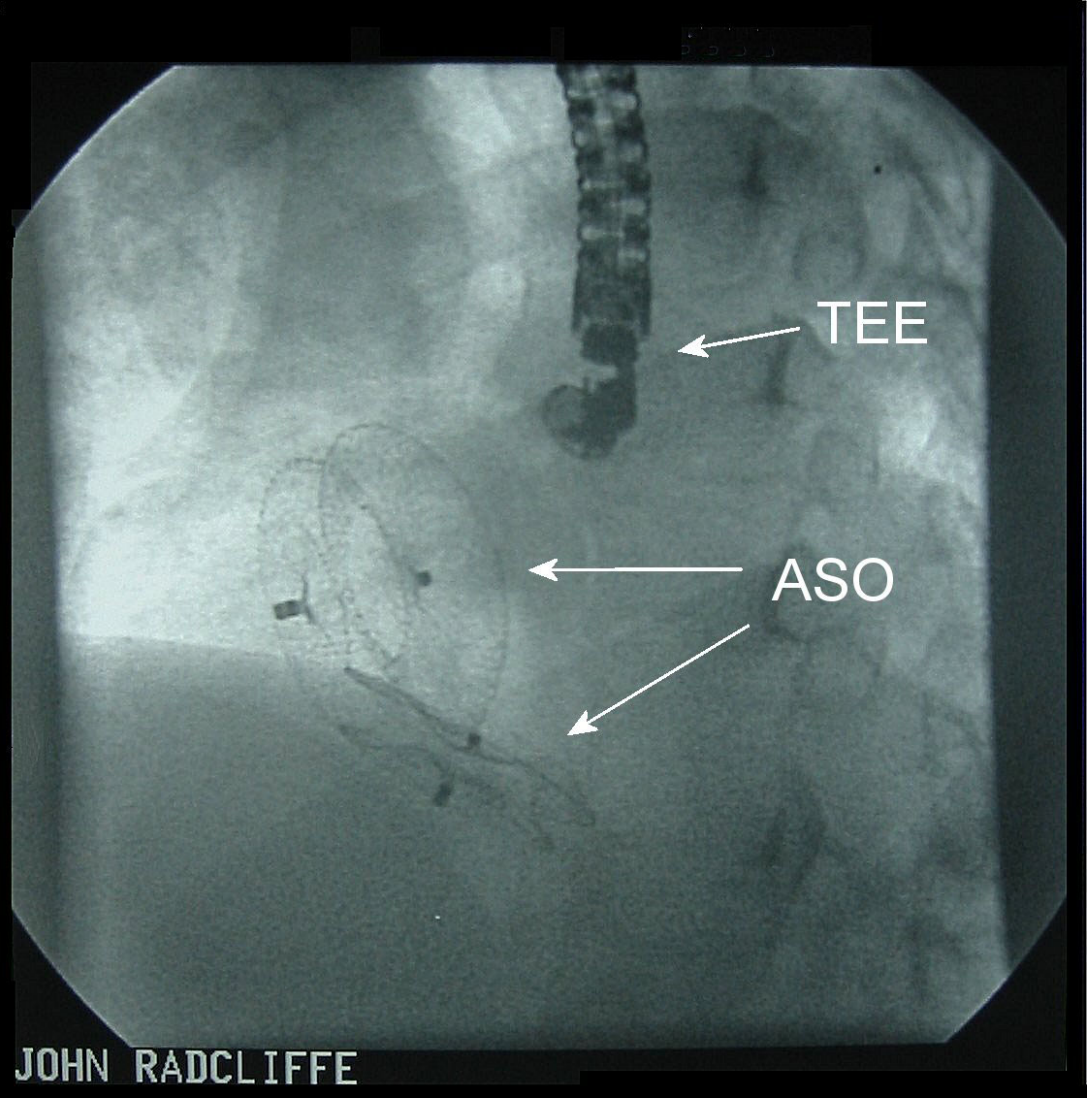
Stored fluoroscopy following placement of the two Amplatzer septal occluders (ASOs). TEE – Transesophageal echocardiography probe.

A 31-year old woman was found to have a secundum ASD during investigations for breathlessness. The defect was estimated to be 10 mm wide on TTE with evidence of right atrial and right ventricular dilatation. Left atrial size was normal. She was referred was further investigation and treatment. Cardiac catheterisation demonstrated a pulmonary to systemic flow shunt of four to one. Peri-procedure TEE revealed that there were two defects; one measuring 24 mm located inferiorly between the inferior vena cava and coronary sinus os with the second defect situated superiorly and measuring 30 mm. The inferior defect was closed using a 24 mm Amplatzer septal occluder. Equivalent chest x-ray radiation dose was 403. The patient was discharged the following day and readmitted three months later for closure of the superior defect. This was performed using a 30 mm Amplatzer septal occluder. The procedure was technical difficult as after deployment of the left sided disc, the device initially lay obliquely in the defect. The final position was satisfactory with no evidence of intra-cardiac shunting (figure 1). There was no interference between the two devices and mitral valve function remained normal. Equivalent chest x-ray radiation dose for the second procedure was 727. The patient remained well and at six month follow-up there was no evidence of residual shunt.

### Case 3

**Figure 2:**
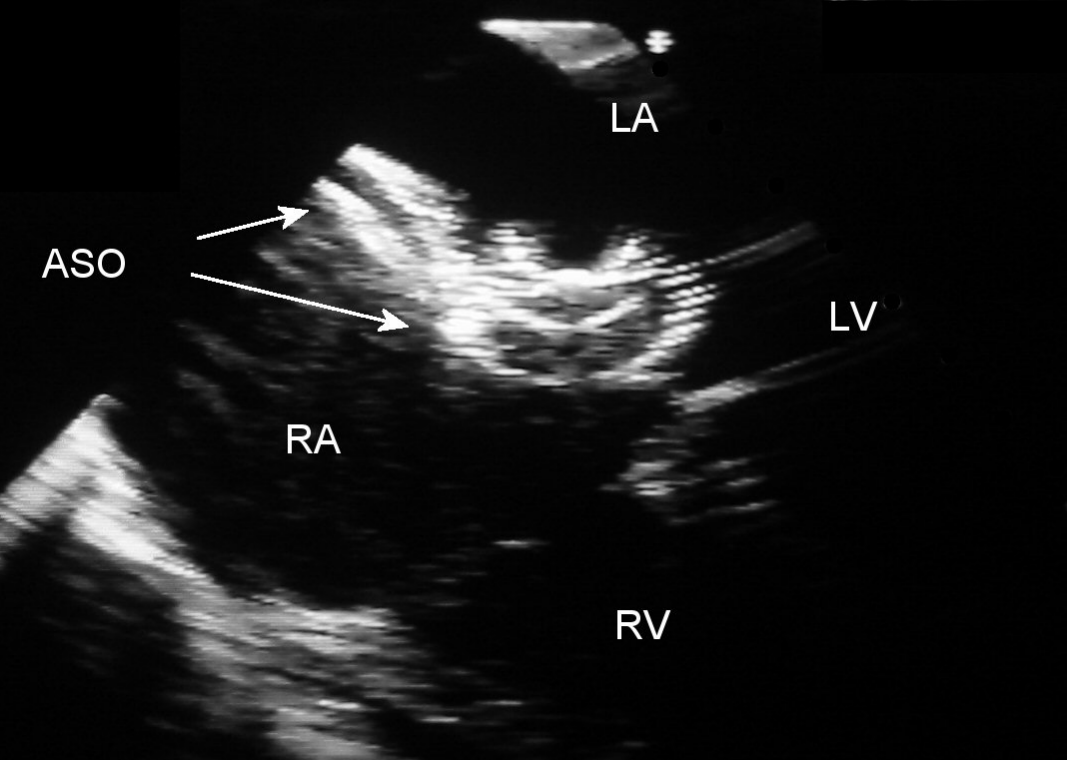
Transesophageal echocardiography four-chamber image following deployment of the two Amplatzer septal occluders (ASO). LA – left atrium, LV – left ventricle, RA – right atrium, RV – right ventricle.

A 30-year old woman was investigated for palpitations and a secundum ASD of approximately 20 mm was diagnosed. TEE suggested that the atrial septum was fenestrated with a small inferior rim of tissue and she was referred for device closure. At cardiac catheterisation it became clear that there were two principal defects, one in the fossa ovalis and the other situated inferior and posterior between the fossa ovalis and the coronary sinus. The superior hole was closed with a 16 mm Amplatzer septal occluder but failed to cover the inferior defect. A 15 mm Amplatzer septal occluder device was subsequently placed successfully across the inferior defect with a stable position (figure 2). Equivalent chest x-ray radiation dose for the procedure was 124. Subsequent follow-up revealed a small left to right shunt between the two septal occluders but further intervention was not considered necessary.

## Discussion

There is considerable morphological variation of secundum-type ASDs. Podnar *et al *reported the echocardiographic findings of 190 patients with isolated secundum ASDs referred for device closure [3]. Twenty four per cent had centrally placed defects but the remaining 144 patients had morphological variations. A deficient superior anterior rim was seen in 42%, a deficient inferior posterior rim in 10%, perforated aneurysm of the interatrial septum was seen in 7.9% and 7.3% of patients had multiple septal defects.

Experience of multiple ASDs closure using more than one Amplatzer septal occluder remains limited [4,5]. In the worldwide report of use of the Amplatzer septal occluder, 3460 patients received a single device but only 45 patients received two devices for multiple ASDs [1]. Cao *et al *reported a series of 22 patients who had two septal occluders implanted simultaneously for multiple ASDs [6]. Closure rate was 97.7% with one device embolisation. In closely positioned multiple defects the septal occluder should be implanted in the largest defect aiming to cover any smaller defects but in widely separated defects more than one device is required. Echocardiographic studies have suggested that patients with multiple ASDs should have a rim of tissue of more than seven millimetres between defects to allow the deployment of two septal occluders [6].

Continuous echocardiographic monitoring is required for device positioning. When TEE is used, patients usually require a general anaesthetic due to the prolonged oesophageal intubation. The development of intracardiac echocardiography now provides an alternative to TEE for device closure. Benefits include more detailed imaging, a reduced need for general anaesthesia, and reduced radiation exposure [7]. In particular, use of intracardiac echocardiography allows clearer visualisation of the inferior atrial septum. Three-dimensional echocardiography may allow more detailed assessment of multiple ASD anatomy and septal occluder positioning.

One question that remains unclear is whether multiple septal occluders should be deployed simultaneously or implanted as staged procedures. Serious complications during single septal occluder implantation is a rare occurrence (less than 0.3% of cases) but it likely that simultaneous deployment will increase the procedure risk [1,6]. When implanting large devices (greater than 20 mm) with little septal separation it is favourable to deploy the larger device and bring the patient back for a further procedure once the device has stabilised.

## Conclusions

There is considerable variation in atrial septal defect anatomy. A small proportion of patients with an ASD have more than one defect and these can be closed using conventional septal occluders under transoesophageal echocardiography guidance. The use of intracardiac echocardiography should allow more accurate device positioning, particularly defects located low in the atrial septum.
